# Identification of dental root canals and their medial line from micro-CT and cone-beam CT records

**DOI:** 10.1186/1475-925X-11-81

**Published:** 2012-10-29

**Authors:** Balázs Benyó

**Affiliations:** 1, Budapest University of Technology and Economics, Department of Control Engineering and Information Technology, Magyar tudósok krt. 2, H-1117 Budapest, Hungary

## Abstract

**Background:**

Shape of the dental root canal is highly patient specific. Automated identification methods of the medial line of dental root canals and the reproduction of their 3D shape can be beneficial for planning endodontic interventions as severely curved root canals or multi-rooted teeth may pose treatment challenges. Accurate shape information of the root canals may also be used by manufacturers of endodontic instruments in order to make more efficient clinical tools.

**Method:**

Novel image processing procedures dedicated to the automated detection of the medial axis of the root canal from dental micro-CT and cone-beam CT records are developed. For micro-CT, the 3D model of the root canal is built up from several hundred parallel cross sections, using image enhancement, histogram based fuzzy c-means clustering, center point detection in the segmented slice, three dimensional inner surface reconstruction, and potential field driven curve skeleton extraction in three dimensions. Cone-beam CT records are processed with image enhancement filters and fuzzy chain based regional segmentation, followed by the reconstruction of the root canal surface and detecting its skeleton via a mesh contraction algorithm.

**Results:**

The proposed medial line identification and root canal detection algorithms are validated on clinical data sets. 25 micro-CT and 36 cone-beam-CT records are used in the validation procedure. The overall success rate of the automatic dental root canal identification was about 92% in both procedures. The algorithms proved to be accurate enough for endodontic therapy planning.

**Conclusions:**

Accurate medial line identification and shape detection algorithms of dental root canal have been developed. Different procedures are defined for micro-CT and cone-beam CT records. The automated execution of the subsequent processing steps allows easy application of the algorithms in the dental care. The output data of the image processing procedures is suitable for mathematical modeling of the central line. The proposed methods can help automate the preparation and design of several kinds of endodontic interventions.

## Background

The shape of the root canal varies from patient to patient, and from tooth to tooth. Severely curved root canals or multi-rooted teeth may pose several challenges in the endodontic treatment. Thus the shape information of root canals can be efficiently used for better intervention planning. Accurate shape information of the root canals may also be used by manufacturers of endodontic instruments in order to make more efficient clinical tools.

The new 3D imaging technologies like cone-beam computed tomography (CBCT) that are available in more and more dental practices show great promise in this field [1]-[4] as they make possible the extraction of the dental root canal shape information. However, the development of root canal shape extraction methods raises a set of challenges due to X-ray dose regulations that cause limited image quality [5]. Moreover, in order to design methods to meet the needs of dental practice the image processing methods should be highly automated.

In order to provide an efficient and effective tool that assists endodontic intervention planning, this research focuses on the automatic recognition of the root and root canals and mathematical description of root canal curvatures. The integration of the image processing steps in novel imaging systems may significantly improve endodontic practice in the near future. In addition, the attempt to automatically locate and classify the root canals may result in decreased chair time for both the patient and the practitioner, reducing clinical burden, effort and cost.

Modern medical imaging devices enable recording several cross sections of the teeth, which can be fed to image processing techniques to extract the shape of the root canal. This problem has been solved several different ways, based on recorded data originating from various imaging tools. Analui *et al*[6] elaborated a geometric approach for modeling and measurement of root canal of human dentition based on stereo digital radiography. Hong *et al*[7] used 2D radiographic images to build up a 3D tooth model, while Endo *et al*[8] turned to ultrasonic imaging and implemented a fuzzy logic based root canal detection. Lee *et al*[9] used micro-CT images and 3D reconstruction software to measure the three-dimensional canal curvature in maxillary first molars via mathematical modeling. Several other 3D dental structure reconstruction systems were elaborated, including Willershausen *et al*[10] who used X-ray images, and van Soest *et al*[11], who applied optical coherence tomography records for 3D structure reconstruction. Germans *et al*[12] presented an imaging system based on virtual reality that can navigate through the reconstructed 3D structure and make measurements concerning the curvature of the root canal.

Recently, further solutions have emerged: Park *et al*[13] proposed a root canal configuration identification method specialized for the first molar, based on micro-CT records. Neves *et al*[14] elaborated a quantitative evaluation technique for caries excavation. Evaluation methods for the morphology of the root canal were given by Verma and Love [15], and Yamada *et al*[16]. The root canal of the incisors were studied by Kaya *et al*[17], who evaluated the changes in the canal’s shape due to aging, and Li *et al*[18], who investigated the effect of manual instrumentation technique on root canal geometry. Recently elaborated modeling tools suitable for the characterization of root canals were given in [19,20].

For further reading in the topic, the reader is referred to the reviews elaborated by Peters [1], Dong *et al*[21], and Swain and Xu [2].

Medial lines of tubular structures are often approximated with 3D curve skeletons [22]. Curve skeleton extraction methodology has a strong foundation. Methods based on thinning or boundary propagation iteratively remove so-called simple points (whose presence does not affect the topology), from the surface of the object. This is generally achieved using a hit-or-miss transform extended to three dimensions [23,24]. Approaches based on distance fields define and compute the minimum distance of each discrete interior point to the surface of the object, an approximate the curve skeleton with the ridges of this distance field [25]. Geometric models generally use a graph-based representation for the approximation of the medial surface or curve of the object [26]. Generalized potential field methods define an internal potential field that differs from the distance field (e.g. electrostatic field generated by placing point charges to all discrete boundary locations [27]), and extract a hierarchical structure composed of critical and saddle points of the field.

3D curve skeletons are composed of loci having at least two closest points on the boundary of the object. This property makes curve skeletons suitable to approximate the center line of the root canal. Curve skeletons preserve the topology of the object, and embody the hierarchy of its components, which is relevant at the detection of bifurcations. In order to suit the needs of dental imaging application, an approach has to be chosen that yields the smoothest curve and shows little sensitivity to slight changes of the object’s boundary. For further details on the topic of 3D curve skeletons, the reader is referred to [22], which is an excellent repository of such methods and their properties.

The general shortcoming of all reported methods that they provide solution only for a single - or at most some subsequent - steps of the root canal identification. The integration of the methods into an automated procedure requires significant enhancement of the algorithms.

This paper introduces predominantly automated image processing procedures for the segmentation of teeth and root canals, and identification of the medial line of the root canal, using a fuzzy chain relation and 3D curve skeletons. Two different procedures will be proposed and validated on micro-CT and one for CBCT records, respectively.

## Methods

### Processing micro-CT records

**Figure 1:**
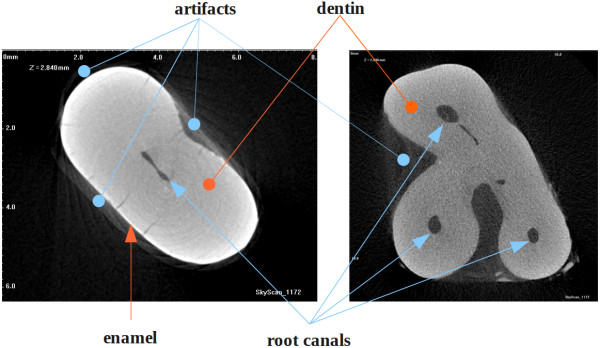
Slices from micro-CT volumes, with typical artifacts.

Dental micro-CT records consist of single channel intensity images, representing high-resolution (1500–3000dpi) scans of parallel cross sections of a certain tooth. A set of images may contain several hundred scanned horizontal planes, which usually are linearly distributed along an axis orthogonal to the scanned planes. The distribution of voxel intensity levels varies from slice to slice, but there are a few rules which most slices obey. In this order, the anatomical structure is reflected by voxel intensities. In normal cases, cross sections contain a light gray spot corresponding to the dentin, usually lighter at its edges (that is because the enamel), possibly surrounding one or more darker regions, which represent the root canal containing soft tissues. The cementum, when visible, is usually somewhat lighter than the dentin. Noise is manifested by granular texture and circular texture visible in the light areas. Figure 1 shows two dental cross sections originating from two different micro-CT records.

The main goal of the proposed image processing procedure is to identify the 3D structure of the root canal built up from the inner darker regions identified from all cross sections. Afterwards, curve that corresponds to the central line is identified and tracked. The detected central line must follow the topology of the root canal, by reflecting its curves and bifurcations.

**Figure 2:**
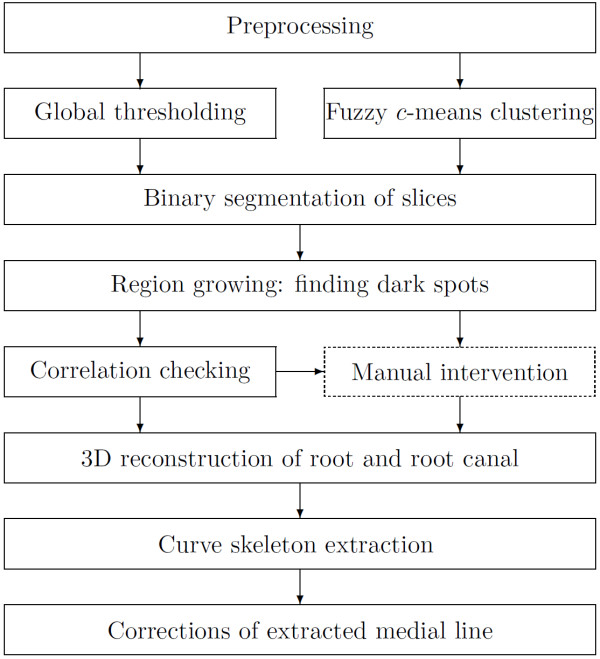
Steps of the proposed algorithm.

Figure 2 exhibits the diagram of the image overall processing procedure proposed. The following subsections discuss the functionality of each box of the diagram.

#### Step MCT1 - Preprocessing

The automatic image segmentation must be preceded by some image enhancement steps. In our application, the following preprocessing steps are employed: 

1. A simple median filter, which reduces the high frequency noise that is most visible in the dentin’s texture.

2. Establishing the region of interest (ROI) by trimming the image: this way we get rid of the dark areas that represent outer space. It is necessary to store the exact coordinates of the ROI.

3. Some basic morphological operators are used to remove texts from the original image and regularize the boundary of the root canal.

After this preprocessing step, the image is ready for segmentation.

#### Step MCT2 - Segmentation in 2D

The final result of the planar segmentation should be a binary image. Even if the image enhancement techniques have already suppressed the disturbing textures, high quality segmentation is obtained by applying a double partitioning.

This step produces two different partitions that are both obtained using the histogram based enhanced version (EnFCM) [28] of the fuzzy *c*-means algorithm [29], which partitions the input slice or volume into a predefined number (*c*) of classes. The first partition is achieved by performing EnFCM on the ROI of the slice, setting the number of clusters to *c*=4. In the followings, this partition will be referred to as local partition, as it is computed from the local data of the slice. The second partition is produced by a simple thresholding operation, using a previously computed threshold *τ*_*global*_that was obtained by EnFCM from the whole data set, using *c*=2 clusters. The latter partition is called global partition of the slice, because it uses the global threshold extracted from the data of the whole volume. Theoretically this would involve computing the global histogram. Instead of that, in order to reduce computation time, the global threshold is estimated using only 2% of the slices, which are linearly distributed along the axis.

The global threshold produces a binary image. The local partition contains 4 different colors, corresponding to the prototypes of the 4 clusters, *v*_1_…*v*_4_. Let us suppose the intensity values are ordered increasingly, that is, *v*_*i* + 1_>*v*_*i*_, ∀*i*=1…*c*−1. The 4 clusters are then separated in two classes, using the threshold *τ*_*local*_=(*v*_*i* + 1_−*v*_*i*_)/2, where $i=\arg\underset{j}{\max}\{v_{i+1}-v_{i},i=1\ldots c-1\}$. In most cases, both binary images are good quality partitions, but there are exceptions, when one of these algorithms fails. In these cases the correct partition must be selected.

#### Step MCT3 - Decision making

To provide an intelligent selection of the correct binary partition, a decision tree has been built based on 250 slices representing above mentioned exceptions. The decision is made in a four dimensional search space, corresponding to parameters: *τ*_*global*_, and *τ*_*i*_=(*v*_*i* + 1_−*v*_*i*_)/2, where *i*=1…3. The output of the tree is the decision whether the local or the global binary partition is the correct one. During the training process, we employed the entropy minimization technique until all the leaves of the tree became homogeneous. After having the decision tree trained, decision making is performed quickly. Finally, a binary image is obtained, where the inner dark regions have to be localized.

#### Step MCT4 - Region growing and selection

The identification of dark spots situated within the light area of the binary image, is performed by an iterative region growing method. As long as there are dark pixels in the segmented image, a dark pixel is arbitrarily chosen and a region is grown around it. Outer space (which is also dark) is obviously discarded, and the detected dark spots are separately stored. Each branch of the root canal, which is present in the cross section, should normally be represented by a single dark region within the slice. Unfortunately, mostly because of imaging artifacts or complex shaped canals, there are some cases, when a single canal branch is manifested by more than one dark region. These cases can be detected automatically, but their treatment sometimes requires manual intervention.

Each dark spot has its center point, which can be computed two different ways: 1) as the center of gravity of the spot; 2) by means of morphological thinning. The center of gravity is easier to compute, but sometimes falls outside the spot. Morphological thinning always gives a quasi-centrally located center point, but entails more computational load.

The automatic selection of detected spots can be performed by several different protocols, which are summarized in Table 1. Protocol P1 can be used in cases of incisor teeth only, when a priori anatomical information makes the presence of a single spot highly probable.

**Table 1:** Implemented spot selection protocols

**Name**	**Description**
P1 - Only one spot	Always extracts the largest dark spot from the slice.
P2 - At most two/three/four	Extracts the second/third/fourth spot if it is present and
	larger than a small threshold size.
P3 - Adaptive	It may extract any number of spots, according to some
	predefined rules that concern the size of different spots.

#### Step MCT5 - Automatic shape regularization

Due to the artifacts present in the original micro-CT records, the dark spot detected in certain slices may contain irregularities. There are several kinds of such cases: some can be treated by automatic regularization techniques, while there are also cases that require manual interaction. For example, a light "island" within the dark spot is easily removable. Strange shaped "peninsulas" can be treated by large masked median filter or morphological opening/closing. There are also cases where the real root canal is detected as several separate dark spots situated very close from each other, which need to be unified. Automatic unification is possible using morphological operations or distance transform.

#### Step MCT6 - Correlation checking

**Figure 3:**
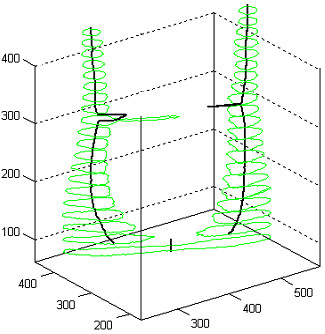
**Checking the correlation of dark spot centers in neighbor slices.** It can reveal the presence of bifurcations (around slice 70) and the need for manual interaction (around slice 310). Both events can be localized and identified automatically.

The accurate segmentation of the micro-CT images may demand manual intervention. Fortunately, the necessity of such steps is visible from the correlation of detected dark spots within adjacent cross sections. Other words, there cannot be a relevant change in the structure found within neighbor slices. Wherever there is a large distance between the center points detected in neighboring slices (see for example Figure 3), either we have a bifurcation, or some intervention is likely to be beneficial. In case of bifurcation, the number of dark spots in the neighbor slices should differ, but correlate with the next neighbor slice for each point. Thus, detecting the need for manual intervention is automated in the proposed process.

#### Step MCT7 - Manual interactions to improve accuracy

The user has the opportunity to change the result of the automatic segmentation within any of the slices. As it was justified in the previous section, the user is advised where the interaction is required. This means that the algorithm automatically detects the cases when the manual intervention is likely to be beneficial and asks for manual intervention. The implemented manual interventions are summarized in Table 2.

**Table 2:** List of implemented manual interventions

**Name**	**Description**
M1	Overrule the decision dictated by the decision tree.
M2	Change the local threshold to any desired value.
M3	Discard some of the automatically detected dark spots.
M4	Unify several dark spots using a parametric active contour
	model (snake) initialized by the user.

#### Step MCT8 - Reconstruct the spatial shape of the root canal

The inner dark spots localized within each slice are put together in space to form a three dimensional object that describes the shape of the root canal. The center line of this object will be searched for using a procedure based on 3D curve skeleton extraction.

#### Step MCT9 - 3D curve skeleton extraction

As mentioned in [22], there are various approximation algorithms for the 3D curve skeleton of voxelized objects. We need to employ such an approach which provides a smooth curve with low amount of branches, and extremely insensitive to zigzagged surfaces. This sort of curve skeleton is reportedly produced by potential field methods. The actual skeleton extraction algorithm implemented into the medial line identification procedure is the hierarchical formulation of the potential field based problem, described in [30].

#### Step MCT10 - Corrections of the extracted skeleton

The 3D curve skeleton accurately handles critical cases like root canal bifurcations, or slices that are far from being orthogonal to the root canal’s direction. Under such circumstances, the curve skeleton is an excellent approximation of the center line. However, at all endings of the root canal, the curve skeleton is either shorter than it should be as the iterative thinning has its effect from every direction, or it has several short branches connected to high curvature points of the surface of the reconstructed 3D object.

To produce an accurate center line with the skeleton extraction algorithm, the divergence parameter of Cornea’s potential field approach must be chosen to be low enough so that the endings of the skeleton towards superficial high curvature points are not present. Further, to avoid the shortened endings of the skeleton, we need to virtually lengthen all endings of the reconstructed tubular 3D object with as many slices (identical to the peripheral one) as necessary. The number of such virtually added slices is well approximated as the shortest radius of the dark spot in the peripheral slice.

Most steps of the algorithm summarized in Figure 2 are performed automatically. Only the box drawn with dotted line comprises any possible manual interactions. This step is not mandatory in simple cases, such as incisor teeth or images with low amount of artifacts.

### Processing cone-beam CT records

**Figure 4:**
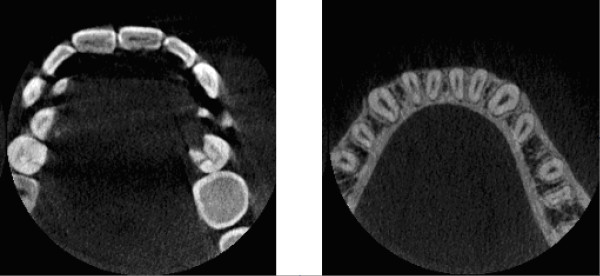
Examples of slices from CBCT volumes.

Cone-beam CT image volumes usually consist of hundreds of parallel equidistant slices, each slice being a single-channel intensity image (some examples are shown in Figure 4). Voxels are isovolumetric, having their size between 100−300*μ*m. At such a low resolution, the root canals frequently have the width of single or a few voxels, and in order to have accurate identification, partial volume effect has to be handled properly. Voxel intensities are recorded as absolute values in Hounsfield units (HU).

#### Step CBCT1 - Image enhancement

The signal-to-noise ratio is in direct proportion with the X-ray dose received by the scanned patient. As the dose should be kept minimal [5], the noise level in the image volume is frequently high. Under such circumstances, an efficient filtering technique needs to be applied to reduce the adverse effect of high frequency noise upon segmentation, without altering or significantly reducing detectable edges. To perform this operation, the context sensitive averaging filter proposed in [31] is employed.

#### Step CBCT2 - Segmentation

Region growing methods usually start from a seed point and grow homogeneous regions around it, by including those neighbor voxels into the region that satisfy a predefined homogeneity criterion. The most frequently used homogeneity criterion is based on voxel intensities, and usually states that a region is homogeneous whenever the standard deviation of voxel intensities within the region is below a predefined threshold. Such segmentation methods frequently have difficulties in noisy environments. High frequency noises can yield several few-voxel regions, while intensity inhomogeneity also hinders the formation of large continuous regions.

The proposed segmentation method is very similar to the classical region growing approach, but there is an important difference that enables it to grow regions beyond noisy voxels. The proposed method is defined as follows.

Let *X*={*x*_1_,*x*_2_,…,*x*_*N*_} be the set of voxels in the image volume, where *N* represents the number of voxels. A fuzzy subset of *X* is defined as a set of ordered pairs:

(1) $$F=\{(x_{i},\mu_{F}(x_{i}))|i=1\ldots N\},$$

where $\mu_{F}:X\to [0,1]$ is called the membership function *F* in *X*. We can define a fuzzy relation in *X* as a fuzzy subset of *X*^2^written as:

(2) $$\Psi =\{((x_{i},x_{j}),\mu_{F}(x_{i},x_{j}))|i,j=1\ldots N\},$$

with $\mu_{\Psi }:X^{2}\to [0,1]$. The so-called *α*-cut of a fuzzy subset *F* is the crisp set:

(3) $$X_{\alpha }^{\left(F\right)}=\{x\in X|\mu_{F}(x)\geq \alpha \}.$$

The fuzzy relation *Ψ*_*α*_ is called a fuzzy link between *x*_*i*_and *x*_*j*_, if:

(4) $$\exists \alpha \in (0,1]:\mu_{\Psi }(x_{i},x_{j})\geq \mathrm{\alpha .}$$

If a fuzzy relation *Ψ*_*α*_holds over a set *X*={*x*_1_,*x*_2_,…,*x*_*N*_}, then we may write *x*_*i*_*Ψ*_*α*_*x*_*j*_ ∀*x*_*i*_,*x*_*j*_∈*X*.

Two elements *x*_*i*_and *x*_*j*_ of a set *X* are *α*-chained, if there exists a sequence of fuzzy linked elements *ξ*_1_,*ξ*_2_,…,*ξ*_*k*_ in *X*, such as:

(5) $$x_{i}\Psi_{\alpha }\xi_{1}\Psi_{\alpha }\xi_{2}\Psi_{\alpha }\ldots \Psi_{\alpha }\xi_{k-1}\Psi_{\alpha }\xi_{k}\Psi_{\alpha }x_{j}.$$

In the proposed segmentation algorithm, two points will be in the same segment whenever they are *α*-chained through neighbor voxels. The only questions that remain are how to define the relation *Ψ* to distinguish voxels belonging to different types of tissues, and which is the right value of *α* that assures a suitable granularity of detected segments?

The goal is to detect a tooth (or a molar) as a single continuous 3D region, and the root canal as another volumetric region inside the tooth. To achieve the above goal, a fuzzy relation for the voxels needs to be defined reflecting the pairwise similarity between the two voxels. Two voxels should be similar if they are close to each other both in physical position and observed intensity.

Similarly to the coefficients of the context dependent filter defined in [31], a fuzzy relation *Ψ* is defined in such a way that it contains the product of two factors:

(6) $$\mu_{\Psi }(x_{i},x_{j})=\delta (x_{i},x_{j})\times \sigma (x_{i},x_{j}).$$

The first factor is a term that depends on the physical distance between the voxels: the closer two voxels are from each other, the more similar they are. The second factor reflects the similarity between the intensity of the two voxels. Here again, equal intensities have the highest similarity, and the larger the difference in intensities, the lower is the degree of similarity.

Two terms are thus defined according to the following rules:

(7) $$\delta (x_{i},x_{j})=\frac{1}{\sqrt{1+\kappa_{\delta }d(x_{i},x_{j})}}.$$

(8) $$\sigma (x_{i},x_{j})=\frac{1}{\sqrt{1+\kappa_{\sigma }|\log\frac{v(x_{i})}{v(x_{j})}|}}.$$

Trade-off parameters *κ*_*δ*_and *κ*_*σ*_enable fine tuning the behavior of the segmentation algorithm. High values of *κ*_*δ*_reduces the proposed method to conventional region growing, while low values enable the algorithm to join regions of similar intensity, which are not physically connected. High values of *κ*_*σ*_determine the algorithm to create regions of piecewise constant intensity, while low ones enable regions to swallow neighbor voxels whose intensity significantly differs from the intensity of the region.

#### Step CBCT3 - 3D object reconstruction

From the segmented image volume, the outer surface of molars and incisors is performed by the corrected version of the marching cube method [32]. This is also employed to reconstruct the shape of the root canal. All surfaces are obtained as triangulated mesh.

#### Step CBCT4 - Medial line extraction

Medial lines of tubular structures are often modeled by 3D curve skeletons [22]. Potential field based methods [27] usually assure smooth curve skeletons of better quality, but they need a much higher resolution of the object. For cone-beam CT volumes, the low resolution of images does not allow employing potential field based curve skeleton extraction methods. As the surface of the root canal is reconstructed as a mesh, it was straightforward to choose to apply the so-called 3D curve skeleton extraction via mesh contraction [33].

## Results

### Accuracy tests

The micro-CT image processing procedure was tested on 25 image volumes that included 17 incisors and 8 molars.

**Figure 5:**
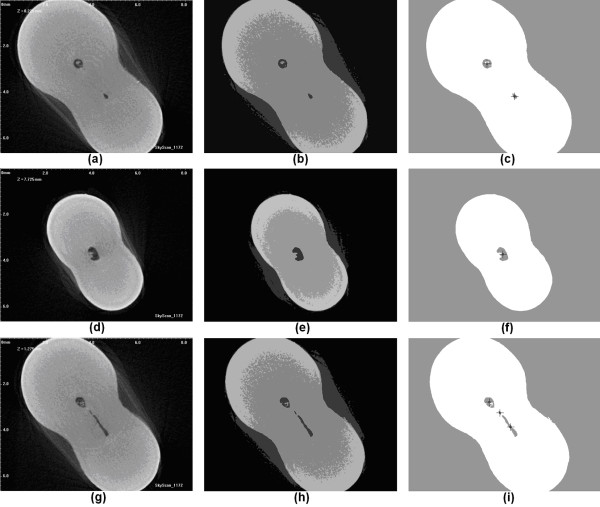
**Detailed view of image segmentation in 2D.** Each row represents a different slice. First column shows the original recorded images; second column presents the clustered images (4 clusters); last column indicates the segmented binary images with detected center points.

**Figure 6:**
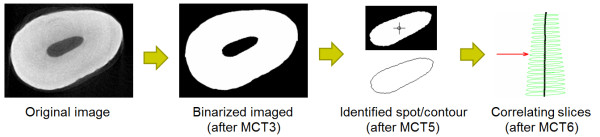
Intermediary results of micro-CT image processing.

Figure 5 exhibits the intermediary results provided by the 2D segmentation. Three cases of various difficulties are presented in the three rows of the image. The first row presents a simple case involving a slice with two dark spots representing two different, easily detectable root canals (there was a bifurcation several slices away from this one). The slice in the middle row manifests an odd shaped dark region, which was successfully detected. The slice presented in the third row shows a difficult case: three different dark spots are present in the segmented images, but they belong to only two different canal branches. This is the case that requires correlation test with neighbors or decision overruling performed by the ANN. Figure 6 shows intermediary results at various points of the process.

**Figure 7:**
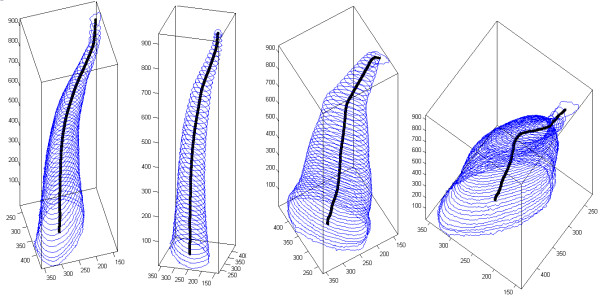
**Various 3D views of a root canal, with the extracted medial line.** Numbers indicate voxels, which are easily convertible to millimeters.

Figure 7 shows four different 3D views of a root canal, together with its detected medial axis. The central line was produced from 944 equidistant slices, segmented in 2D with binary separation using the global optimal threshold.

The CBCT volume processing procedure was tested on 36 image volumes that contained incisors and molars in equal number. The proposed algorithm requires a single interaction: the user is asked to mark the tooth (incisor or molar) desired to be segmented. The volume of interest is then processed automatically.

**Figure 8:**
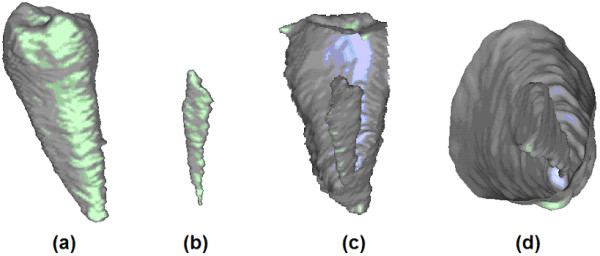
Various reconstructed views of an incisor and its root canal: (a) shape of the incisor; (b) shape of the root canal; (c)-(d) sectional views.

**Figure 9:**
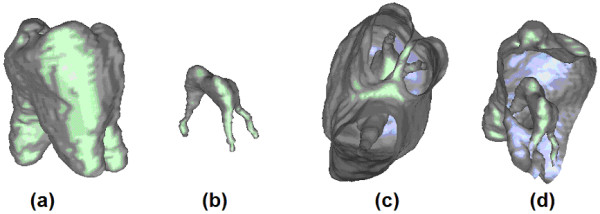
Various reconstructed views of a molar and its bifurcated root canal: (a) shape of the molar; (b) shape of the root canal; (c)-(d) sectional views.

The surface of segmented teeth and root canals are produced as mesh. After the automatic processing, the user may also visualize sectioned views of the tooth. Figure 8 exhibits some views produced in case of an incisor, while Figure 9 shows the result provided in case of a molar with three branches in the root canal. In both these figures, part (a) shows the shape of the tooth, (b) the shape of the root canal, represented at the same scale, in vertical position correlated with (a). Images shown in (c) and (d) are various sectioned views of the tooth and root canal, visualized together.

**Figure 10:**
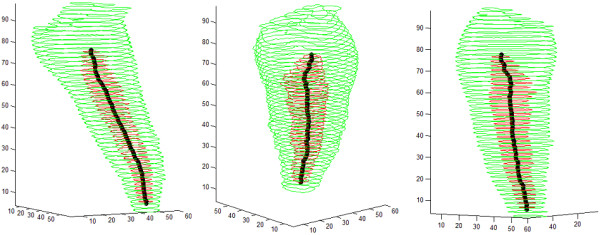
Various 3D views of a root canal having no bifurcation, with the extracted medial line.

Figure 10 presents the extracted central line in case of an incisor, together with the shape of the root canal and the shape of the tooth. All three images represent the same tooth, visualized from different angles. Sections of the tooth are shown using elevated contour plots, green contours indicating the shape of the tooth, and red ones the shape of the detected root canal. Indicated coordinates represent distances in voxels, which are easily convertible to millimeters.

**Figure 11:**
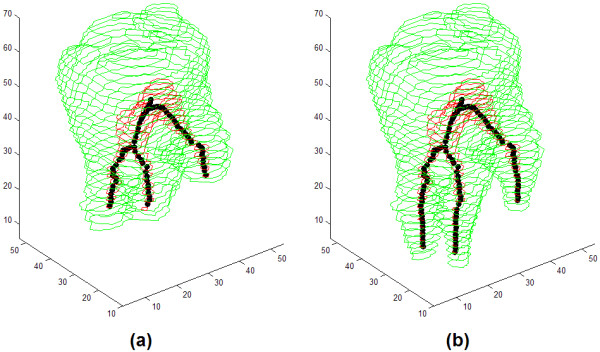
3D views of a molar with its bifurcated root canal and the extracted medial line: (a) before and (b) after manual intervention.

Figure 11 shows two different versions of a segmented molar, using the same representation conventions: the image on the left side, having shortened roots and root canals, is obtained automatically, while the image on the right is the correct segmentation obtained after manual intervention. The simple intervention was needed to inform the algorithm that four distinct volumetric regions that were automatically detected, in fact belong together to form a whole molar.

The accuracy of the segmentation is usually better in case of incisors than in case of molars, because the latter have more details to identify. The main difficulty comes from the low resolution of the CBCT imaging technology, which makes some structures have tiny sizes, sometimes under the unit size of a voxel. The identification of the medial line is efficient and accurate: as the width of the root canal hardly ever exceeds 10 voxels, the mesh contraction algorithm finishes the extraction of the curve skeleton in 7-8 iterations. The identified skeleton is smooth, centrally located, and its bifurcations are suitable to model the actual shape of root canal branches.

Both proposed procedures can automatically and accurately process more than 90% of the recorded image sets, while the rest of the cases need serious amount of manual interaction. The MCT procedure was tested on 25 image volumes. In case of two molars, several uncorrelated neighbor slices were found, where the necessity of manual interaction could not be accurately detected. In the other 23 volumes, a total number of 227 (out of 23391) slices were advised for expert inspection, but only 119 of them required actual intervention. This amount of false positives is acceptable. The correlation between the automatic decisions made by the developed algorithm and the expert validating the algorithm is shown in Table 3.

**Table 3:** Accuracy details of automatic detection of the need for manual intervention in the case of CBCT records

		**Expert evaluation**		
		**No need for Intervention**	**Intervention needed**
**Automatic detection**	**No need for intervention**	23150	14
	**Intervention needed**	108	119

Out of 36 image volumes used for testing the CBCT procedure, 29 led to correct segmentation and identification of the root canal without needing any automatic interaction. In case of 7 molars, manual interaction was needed, being able to solve 4 of them, similarly to the case exhibited in Figure 11. The quantitative validation results of the two developed algorithms are shown in Table 4.

**Table 4:** Quantitative results of validation

**Description**	**Micro-CT**	**CBCT**
Total test image volumes	25	36
Successfully processed image volumes	23	33
Overall success rate	92.0%	91.7%
Overall success in incisors	100.0%	100.0%
Overall success in molars	75.0%	83.3%
Minimum processing time	288 sec	0.69 sec
Average processing time	341 sec	0.93 sec
Maximum processing time	490 sec	1.61 sec

The proposed procedures provide useful information for mathematical description of the root canal’s shape. The medial line can be successfully approximated by spline curves, as described in [34,35].

### Efficiency

Using a i5-processor PC, the processing of a micro-CT slice in 2D lasts 0.2-0.3 seconds, while a central canal reconstruction is performed in less than a second. The extraction of 3D curve skeleton representing the canal’s medial axis requires 10-15 seconds, depending on the number of voxels in the canal’s volume. On the other hand, having roughly 0.5−1.5×10^6^ voxels in the volume of interest, the identification of a manually selected tooth in a CBCT volume automatically performs in less than a second.

## Discussion

The key advantage of both algorithms is the high degree of automatic execution, and the ability of automatically detecting the necessity of manual interventions. In case of micro-CT records, the latter feature stems from checking the correlation between neighbor slices. Wherever the correlation is weak, it can be because of: (1) bifurcations of the root canal; (2) mistaken segmentation in 2D. The number, size and position of detected sections of the root canal, situated within the investigated neighbor slices, are the main data for the decision. In case of CBCT records, steps CBCT2 identifies piecewise continuous volumetric regions that belong to the dentin, which are reconstructed to form the tooth in Step CBCT3. There are cases, when unifying these volumetric regions is not obvious. In such cases, without manual interventions, we may lose the inferior, narrow part of the root canal branches. However, manual intervention can help us out in these cases as well (see Figure 11).

An essential tool in the MCT procedure is the decision tree implemented in Step MCT3, responsible for the correct outcome of the 2D segmentation. The training data set consisting of 250 images, was selected from seven image volumes, through an automated process. Those images were selected as suitable candidates, for which the two EnFCM-based segmentations (*c*=2 and *c*=4) did not correlate in any combination of the classes. Using the test image set of 25 volumes of approx. 1000 slices each, the decision learnt from 250 training images proved acceptable, in the sense that less than 0.1*%*of the slices needed manual intervention due to the wrong decision of the tree.

The developed procedures certainly have some limitations, too. The reduced amount of image data originating from a single CT imaging system, which was used for the creation of the procedures, certainly could not cover all typical root canal deformations. A larger amount of images would definitely make the system more stable and its decisions more robust.

## Conclusions

We have proposed and implemented two complex image processing procedures for detecting the center line from dental micro-CT and CBCT records. Both procedures work predominantly automatically, providing the opportunity for the user to improve the outcome using some optional manual interventions only where needed.

The proposed image processing procedures are validated on real micro-CT and CBCT images. Over 90% of the test data set was segmented and identified automatically and correctly. The identified center lines are accurate and suitable for further mathematical modeling (e.g. spline curve fitting). Thus, this research has created and validated the image processing systems and corresponding image processing methods to efficiently assist common dental interventions.

## Abbreviations

CT: Computed Tomography; CBCT: Cone-beam Computed Tomography; FCM: Fuzzy c-means; 2/3 D: two/three dimensional, EnFCM: Enhanced Fuzzy c-means.

## Competing interests

The authors declare that they have no competing interests.
